# Development of freeway-based test scenarios for applying new car assessment program to automated vehicles

**DOI:** 10.1371/journal.pone.0271532

**Published:** 2022-07-21

**Authors:** Woori Ko, Sangmin Park, Sungho Park, Harim Jeong, Ilsoo Yun

**Affiliations:** 1 Department of Transportation System Engineering, Ajou University, Suwon, Republic of Korea; 2 Department of Road Transport Research, Korea Transport Institute, Sejong, Republic of Korea; Tsinghua University, CHINA

## Abstract

As automated driving technology continues to develop, studies are being conducted to develop various scenarios for assessing the functional safety, failure safety, and mobility of automated vehicles (AVs). As the commercialization of AVs progresses, it is necessary to develop a set of test scenarios for new car assessment programs (NCAPs), so as to provide information on the safety and reliability of AVs to consumers. To provide valuable information regarding newly emerged AVs to consumers who are willing to purchase them, it is necessary to derive specific and well-defined test scenarios based on the safety-in-use. Accordingly, to apply NCAPs to AVs, this study established test scenarios targeting freeways where AVs were expected to be commercialized. To this end, based on freeway traffic accident data and opinions of traffic safety and AV experts, we derived possible dangerous situations when an AV is maintaining a lane on a freeway. Functional scenarios were defined based on the derived dangerous situations. The priority of the defined functional scenarios was set using the analytic hierarchy process (AHP). Accordingly, this study presents a logical and concrete scenario construction methodology for deriving the ranges and values of test parameters for functional scenarios.

## 1. Introduction

As various organizations such as automobile original equipment manufacturers (including Mercedes-Benz, BMW, GM, Hyundai Motors, and Tesla) and IT companies (such as Google, Apple, and Uber), universities, and start-ups accelerate the development of automated vehicle (AV) technology, AVs are getting closer to commercialization [1–3]. As interest in AVs increases, studies are being conducted to develop various scenarios for evaluating their functional safety, failure safety, and mobility [4, 5]. Most of these studies are focused on the development of scenarios for assessing the functional safety of AVs in relation to development evaluation and audits.

However, as AV commercialization approaches, there is an increasing demand to provide safety assessment results for AV to consumers through the new car assessment programs (NCAP). An NCAP is a system for inducing rational purchases by delivering safety assessment results for automobiles to consumers. In an NCAP, tests such as head-on collision, side impact, and rollover are performed according to a protocol to evaluate the safety of a vehicle, and the results are scored and then disclosed to consumers. Studies related to the passive and active safety of automobiles must have high accuracy and reliability, as they are directly related to human life. Currently, NCAPs are evaluating advanced driver assistance system (ADAS)-related functions in autonomous driving technologies and are expected that will soon conduct safety assessments of AVs [6, 7]. In the Korean NCAP(KNCAP), like the Euro NCAP, the functions of ADASs that comply with the Society of Automotive Engineers (SAE) Level 2 requirements are being tested. In order to meet these demands for safety information of AVs, scenario-based evaluation is required because the automated driving system is much more complex than ADAS.

Therefore, this study aimed to derive a safety assessment scenario development methodology for testing AVs in the KNCAP, targeting Level 3 AVs as defined by SAE [8]. To provide valuable information regarding newly emerged AVs to consumers who are willing to purchase them, it is necessary to derive specific and well-defined test scenarios reflecting actual driving situations. From traffic accident data related to AVs provided by the California DMV, it can be observed that a large number of traffic accidents are caused by vehicles other than AVs [4]. Therefore, it is necessary to derive the dangerous situations that an AV will encounter when maintaining a lane on freeways, and to determine whether the AV responds appropriately and defensively in those situations. Scenarios created only based on the experiences of experts have clear limitations in reflecting the various situations potentially occurring on real roads. To determine the reliability of AV safety assessment results and consumer acceptance, realistic and specific test scenarios considering the dangerous situations that drivers experience in daily life are required. In this study, this perspective is referred to as "safety-in-use." Various test scenario development methodologies have been suggested in the PEGASUS project scenario, "safety assurance KUdos for reliable autonomous vehicles" (SAKURA) project, etc., but the application of safety-in-use-based approaches has been relatively limited [9–13]. Therefore, it is expected that creating scenarios based on real traffic accident data (and thereby reflecting actual situations) will be more effective. By deriving AV test scenarios related to freeway lane maintaining using freeway traffic accident data, KNCAP can deal with realistic and specific AV assessment scenarios.

In this study, the spatial range for the AV test scenario development was set to maintaining the AV within the main lanes among the freeways in Korea, except for on ramps and tollgates. In this study, the freeway traffic accident data from general automobiles and expert opinions were considered together to develop the scenarios. The temporal range of the traffic accident data was from 2012 to 2014. The temporal range of traffic accident data was relatively old, as the Korean National Police Agency (KNPA) does not disclose recent traffic accident data. In addition, this study built the AV test scenarios based on the functional, logical, and concrete scenario concepts presented by the PEGASUS project [9, 10].

Section 2 examines related trends and studies. Section 3 defines the need for AV tests, and describes the direction for the safety assessment. Section 4 details the development of the test scenario derivation methodology and scenario framework for testing AVs, and the development of test scenarios according to the framework. Section 5 presents the results from the test scenarios. Section 6 presents the conclusions and directions for future development.

## 2. Literature review

### 2.1 Automated Vehicle (AV) test scenario development trend

The PEGASUS project is a project for the establishment of generally accepted quality criteria, tools and methods, as well as scenarios and situations, for the release of highly automated driving functions. It is organized under the leadership of the German Federal Ministry for Economic Affairs and Energy [9, 10]. In the PEGASUS project, the types of functional, logical, and concrete scenarios are defined according to the level of abstraction of the automated driving evaluation scenario. Each scenario refers to the concept definition, system development, and test stages. A functional scenario presents conceptual explanations of roads, environments, and situations. A logical scenario includes the parameters and ranges necessary for scenario expression. Finally, in the concrete scenario, the reproducibility of the experiment is guaranteed by presenting specific parameter values [14]. In addition, as shown in Table 1, the PEGASUS project defines a six-layer model classified according to the characteristics of each parameter, thereby providing a systematic explanation of the experimental scenario [10, 15].

**Table 1:** Six-layer model defined by the PEGASUS project. Source: [15].

Layers		Details
1	Road-Level	Geometry, topology
		Quality, boundaries (surface)
2	Traffic infrastructure	Boundaries (structural)
		Traffic signs, elevated barriers
3	Temporary manipulation of L1 and L2	Geometry, topology (overlaid)
		Time frame > 1 day
4	Objects	Static, dynamic, movable
		Intersections, maneuvers
5	Environment	Weather, lighting and other surrounding conditions
6	Digital Information	V2X information, digital map

In 2016, the National Highway Traffic Safety Administration (under the U.S. Department of Transportation) presented guidelines for evaluating the design, development, and operation of AVs through the announcement of the “Federal Automated Vehicles Policy” [16]. In 2017, it announced “Automated Driving Systems: A Vision for Safety 2.0,” which presented 12 major safety factors based on a parliamentary hearing and feedback from automated driving experts [17]. Recently, the automated driving guidelines have been continuously updated, e.g., through the announcement of “Preparing for the Future of Transportation: Automated Vehicles 3.0” in 2018 and “Ensuring American Leadership in Automated Vehicle Technologies: Automated Vehicles 4.0” in 2020 [18, 19].

The SAKURA project proposes an engineering framework for AV safety assessment scenario development in Japan [11]. The proposed framework first defines the operational design domain (ODD) in consideration of safety goals, then develops test scenarios and conducts tests [11–13]. The test scenario development is narrowed down to a range corresponding to preventable conditions after defining parameter items and foreseeable conditions based on actual data [11].

The CETRAN (Excellent Center for Autonomous Vehicle Testing and Research) is a project to develop standards and behavioral capabilities to promote the application of autonomous vehicles in Singapore’s urban environment. Therefore, CETRAN develops a test methodology for adopting and introducing technologies related to autonomous vehicles. Regarding scenarios, a data-based approach is presented, focusing on qualitative explanations of scenarios, and scenario categories are classified by introducing appropriate tags [20]. The scenario tag describes the scenario category by defining features such as ego, actor, road type, road layout, weather, etc. In CETRAN, 67 scenario categories were derived [20].

The Association for Standardization of Automation and Measuring Systems (ASAM) promotes the standardization of tool chains for use in vehicle development and testing [21]. The ASAM consists of auto manufacturers, suppliers, and service providers world-wide; it receives ideas from members, and turns them into projects. The projected ideas are made into standards so that they can be interconnected with development process tools and data can be exchanged smoothly [21]. Among the various standards developed by ASAM, OpenSCENARIO defines a file format for describing the dynamic contents of driving and traffic simulators [22]. OpenSCENARIO is used to test, verify, and certify the safety of ADASs and AVs by defining concrete descriptions of scenarios and developing multiple levels of abstraction for scenario descriptions [23–25].

Among WP.29, the "Working Party on Automated/Autonomous and Connected Vehicle" introduced a new assessment/test method (NATM) master document for evaluating automated driving systems (ADSs). The NATM builds scenario catalogs for situations requiring safety assessments, and validates them through simulation/virtual testing, track testing, real-world testing, audit/assessment, and in-service monitoring and reporting; this type of approach is called a "multi-pillar approach" [26]. The NATM master document also introduces functional scenario examples for freeway driving [26].

### 2.2 Prior studies

Yang et al. [27] proposed an automatic emergency braking (AEB) model for avoiding collisions. To evaluate the proposed AEB model, car-to-car rear braking and car-to-car rear moving scenarios were used for Euro NCAP’s AEB test. The proposed AEB model recognizes the intention of the front driver as a backpropagation-hidden Markov model, and then transmits it to the rear vehicle through the Internet of vehicles, so that the rear vehicle dynamically changes its braking strategy. As a result of the evaluation, the proposed AEB model provided more effective braking than an existing AEB model with fixed parameter values, and safely avoided collisions.

Gelder et al. [28] proposed a scenario-based real-road approval assessment procedure for the safety of AVs from the perspectives of applicants, assessors, and authorities. According to the proposed procedure, after the applicant designs and develops the AV, the safety test scenario and test results are provided to the assessor. The assessor assesses the completeness of the tests and checks the results. The authority sets the ODD and dynamic driving task requirements for the AV, and decides whether to approve the AV for real road driving.

So et al. [29] presented a methodology for developing AV test scenarios. Based on big data technology, the descriptions of traffic accident data were analyzed with an automated analysis program, and AV test scenarios were derived using a combination of 19 frequently mentioned keywords. Subsequently, to verify the scenarios derived using the big data-based methodology, a derived scenario was evaluated via manual investigation. The results from the approaches of the big data-based scenario and manual investigation scenario were consistent, and reliability was accordingly secured. In addition, it was confirmed that the big data-based scenario was efficient, as it could be built in a shorter time than a manual investigation.

Takács et al. [30] presented a review of Level 2–3 ADSs. Currently, the Euro NCAP is the most widely used automated driving assessment program. The Euro NCAP introduced ADAS function evaluation in 2010, and began testing new cars with Level 2 functions in 2018. The study suggested that to apply a Euro NCAP Level 2 scenario to a Level 3 ADS, the scenario needed to be modified so that the sensor improvements and appropriate maneuvers during fallback could be considered.

Erdogan et al. [31] indicated that existing scenario derivation studies based on expert opinions have apparent limitations in reflecting real situations. They claimed that a scenario-based evaluation approach is required to test the safety of AVs, and that an approach to extracting scenarios from actual road driving data can help in overcoming these limitations and developing various scenarios. In addition, three methods were proposed for extracting scenarios from the time-series states of real-world measurement data, e.g., using machine-learning algorithms for multiple classifications.

As a result of reviewing the related studies, it can be seen that many studies have proposed diverse methods for creating scenarios related to the assessment and auditing of AVs, but it is difficult to find studies on developing test scenarios for applying NCAPs to AVs. As AV safety is a matter directly related to life, research is needed to develop NCAP evaluation scenarios for AVs to increase such safety. Therefore, unlike previous studies, this study intends to establish scenarios based on safety-in-use and examples of situations threatening AVs when driving, and to introduce a spatial scope into freeway and NCAP assessments.

## 3. Need and assessment direction for applying New Car Assessment Program (NCAP) to AV

### 3.1 Need for applying NCAP to AV

An NCAP evaluates the safety of vehicles sold in the market and aims to provide consumers with an easy-to-understand result [6, 32]. As the commercialization of AVs progresses, consumers are curious about the safety level of newly emerged AVs. In addition, as many consumers have never experienced AVs, if safety information on AVs is provided, it is expected that they will purchase AVs after reference to the information.

In the case of the KNCAP, it provides safety information by assessing the ADASs of Level 2 vehicles, and research on how to assess Level 3 AVs is currently in progress. In addition, if the NCAP for Level 2 vehicles is centered on functional safety, the evaluation of level 3 AVs needs to focus on comprehensive driving safety, take-overs, and minimum risk maneuvering. Therefore, it is considered that the biggest difference in assessments for Level 2 vehicles with ADAS and Level 3 AVs is in the test scenarios. Accordingly, to systematically and objectively evaluate AVs in the KNCAP, it is necessary to derive test scenarios, after establishing a test scenario development direction reflecting the domestic traffic conditions.

### 3.2 Assessment direction for applying NCAP to AV

In Korea, there are “Rules on the Performance and Standards of Automobiles and Auto-Parts,” called safety standards, and “Enforcement Rules for Performance and Standards of Automobiles and Auto-Parts," called enforcement regulations. The safety standards suggest the minimum levels of safety and performance required from automobiles [33]. The enforcement regulations provide the detailed standards and test protocols necessary for the implementation of the safety standards [34]. In 2020, the safety standards and enforcement regulations related to freeway lane keeping for Level 3 AVs in Korea were revised.

Nevertheless, in an NCAP, it is necessary to implement a more challenging assessment than those for the safety standards and enforcement regulations, so as to promote safety technology development, rather than minimum safety guarantees. In the case of enforcement regulations, it is generally simply evaluated whether the AV functionality works under normal driving conditions. Therefore, it is considered that the KNCAP should be differentiated from safety standards and enforcement regulations, e.g., by comprehensively assessing the defensive driving abilities of AVs in more realistic and challenging situations. In other words, it is considered necessary to develop NCAP test scenarios focusing on situations that drivers actually experience while driving on freeways, such as a situation that conflicts with that of another vehicle on the freeway, or a dangerous situation where the next action of the AV must be quickly decided. In addition, the actual situation needs to be considered in terms of the transportation facilities. For example, it is necessary to evaluate whether an AV drives well not only in a normal lane but also in a partially damaged lane.

## 4. NCAP test scenario development methodology

### 4.1 Overview

In this study, in order to develop the KNCAP AV test scenarios, the methodology of the most recent trends, PEGASUS project, SAKURA project, and CETRAN was benchmarked through literature review. Especially, the PEGASUS project’s functional, logical, concrete scenario development method, concepts and six-layer format were benchmarked, and data suitable for the Korean situation were collected and analyzed to develop scenarios suitable for Korea. A description of the benchmarked methodologies was written in the Literature Review part of Section 2. Section 4 deals with the development methodology of the newly derived scenario.

### 4.2 NCAP test scenario development framework

**Fig 1:**
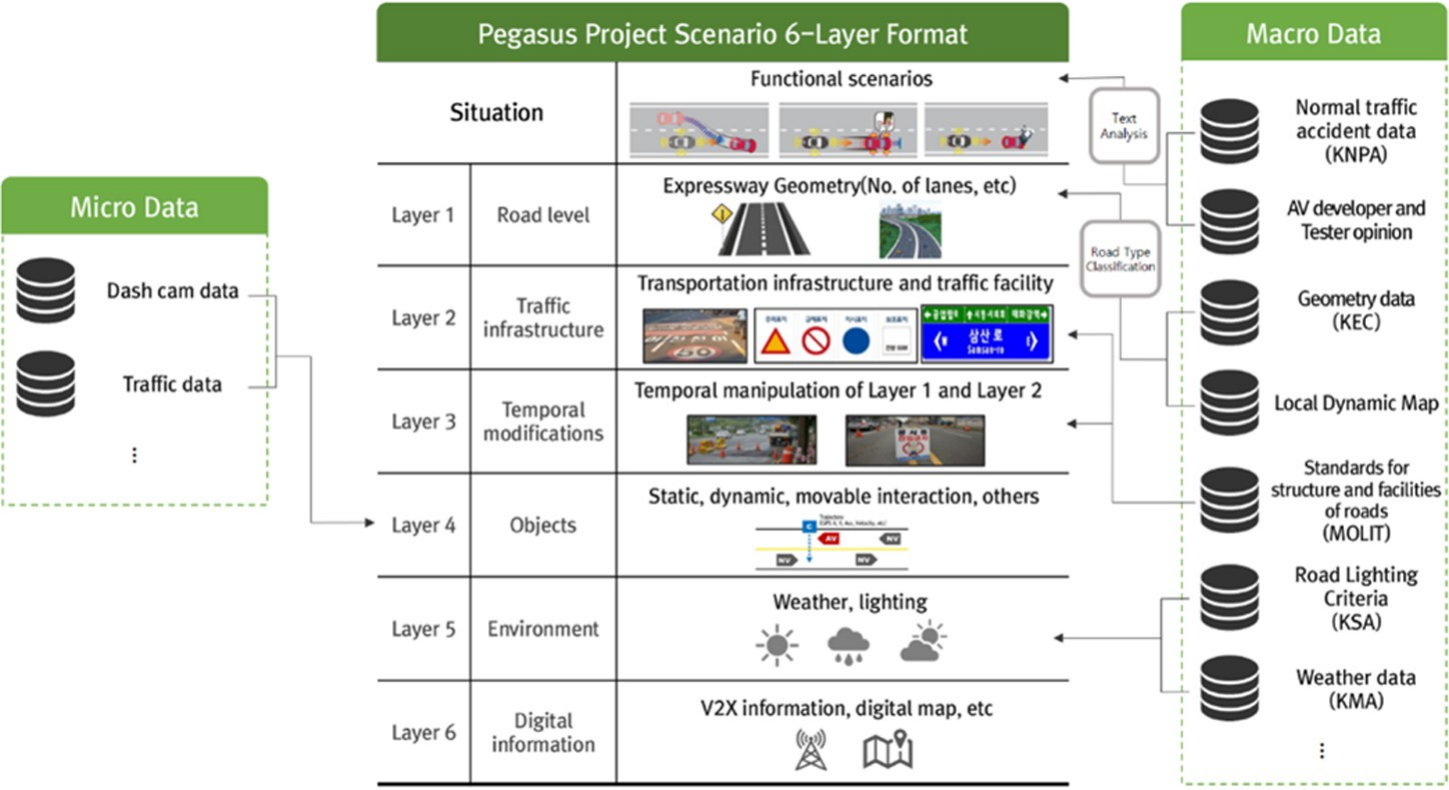
Automated vehicle (AV) assessment scenario framework.

This study presents an AV test scenario development methodology that can be used in the KNCAP. Based on the scenario definition method of the PEGASUS project and its six-layer format, an internationally operable and sustainable scenario development framework was established. The scenario development framework aimed to promote a systematic and objective assessment of freeway lane-keeping performance. As can be seen in Fig 1, this study built scenarios with traffic accident data for general automobiles, as there are currently not enough ADAS or AV traffic accident data in Korea. However, it can be supplemented by adding (and/or by replacement with) traffic accident data of ADASs or AVs in the future. Scenarios were constructed with two tracks, consisting of (1) an approach based on the opinions of experts in AV and traffic safety, and (2) an approach using traffic accident data. In addition, the parameters necessary for scenario development were collected from macroscopic and microscopic data sources, as shown in Fig 1. Macroscopic and microscopic data were defined in scenario derivation aspect as data representing the overall road traffic situation and the route-based traffic situation of individual vehicles, respectively. Therefore, macroscopic data is an aggregation of microscopic data and the average value is mainly used to extract the entire road situation. While, microscopic data mainly used individual vehicle driving trajectories. A detailed description is provided below.

### 4.3 Data collection

To assess the safety of AVs, it is best to use AV traffic accident data. However, the existing AV traffic accident data suitable for domestic traffic conditions remains insufficient. Therefore, the KNPA’s traffic accident data for general automobiles were used. The KNPA’s traffic accident data comprised 58 pieces of information, including those concerning the time, place, accident type, vehicle model, and descriptions. A total of 10,364 cases of freeway traffic accidents were collected between 2012 and 2014. Among them, to extract only the spatial range set in this study, 8,225 traffic accidents were extracted by spatial calculation using QGIS 3.10, and traffic accidents that occurred on ramps and tollgates were excluded. Of the 8,225 traffic accident data, 6,951 traffic accident data were ultimately selected after excluding traffic accidents caused by drunk driving. Using data from the 6,951 traffic accidents, we derived the dangerous situations that AVs will experience on actual freeways in the future.

In addition, information such as the design speed, lane width, and curvature was collected using freeway geometry data provided by the Korea Expressway Corporation (KEC). Information such as the lane size was collected using the rules on road structure and facility standards from the Ministry of Land, Infrastructure, and Transportation (MOLIT). The road lighting standard information was provided by the Korea Standards Association (KSA). For freeway environmental condition data, information on the domestic precipitation and fog was provided by the Korea Meteorological Administration (KMA). Finally, information concerning, e.g., accident locations, moving distances, vehicle speeds, and time-to-collision (TTC) was collected through an image analysis of dash-cam data owned by domestic insurance companies.

### 4.4 Functional scenario development methodology

The functional scenario is a stage for presenting conceptual explanations of roads, environments, and situations; hence, the description of the situation is key. In this study, the description of the situation was derived using the "descriptions" from among the freeway traffic accident data of the KNPA, and the opinions of experts in AVs and traffic safety. The descriptions followed a format such as "#1 vehicle did not notice that #2 vehicle had stopped, so #1 vehicle struck the rear of #2 vehicle with the front bumper of #1 vehicle." The description described the traffic accident process in detail, such as the cause of the accident, how the collision progressed, and which parts between vehicles collided. The accident process was described in the text; therefore, the text of the situation information was analyzed using a text mining technique. As a result of the text mining process, traffic accidents were classified into eight accident categories. Eight accident categories are traffic accidents while driving at high speed, while driving at low speed, traffic accidents owing to stationary object, owing to cut-in, owing to cut-out, traffic accidents caused by reverse vehicle, other traffic accidents, and not applicable traffic accidents. The frequency of occurrence was calculated according to the classified categories, and possible dangerous situations were derived through a detailed analysis. By listening to the opinions of experts in AVs and traffic safety, additional dangerous situations potentially occurring for AVs were identified. The derived dangerous situations were reviewed as functional scenarios for the NCAP.

### 4.5 Priority derivation methodology

The constructed functional scenario proceeds to a logical scenario and concrete scenario, and the amount of information exponentially increases. It is practically impossible to evaluate all scenarios; therefore, it is necessary to construct representative scenarios through verification and priority derivation. To this end, experts in AVs and traffic safety were consulted to establish the priority of the functional scenarios based on an analytic hierarchy process (AHP). The priority-building procedure for the functional scenarios was as follows.

1. The priority evaluation items for the AHP are derived, as shown in Table 2.
2. The priority evaluation items are standardized to a value between 0 and 1 according to the survey results of the experts and are used as weights.
3. Experts indicate their survey scores on a 5-point Likert scale for each constructed functional scenario.
4. The comprehensive scores are calculated by multiplying the score for each scenario by the calculated weight.
5. The higher the total score finally derived, the higher the priority.

**Table 2:** Scenario priority evaluation items.

Evaluation Items	Contents
Ease of new car assessment program (NCAP) test	Scenario must be practically evaluable, and the experiment must be easily reproduced for assessment.
Traffic safety effectiveness	Scenario should contribute to the reduction of related accidents.
Technical challenge	Scenario should induce vehicle manufacturers to develop autonomous driving technology.
Clarity of evaluation results	Purpose of the experiment, contents and assessment results must be clearly and easily communicated to consumers.
Differentiation from existing assessments	Content and purpose of scenario should be different from the existing vehicle safety standards and test method.

### 4.6 Logical and concrete scenario derivation methodology

In the logical scenario, it is essential to select the evaluation-related parameters based on the built functional scenarios, and to derive the range of each parameter value. In this study, the logical scenario was used to analyze the collected data and define the parameters and ranges potentially affecting the AV maneuvers in the situation, based on the six-layer format. The reason for using the six-layer format was that the parameters to be considered in a scenario could be easily organized according to the characteristics of each parameter.

In the concrete scenario, it is essential to present the parameter values to be used in the test, so as to ensure the reproducibility of the experiment. Accordingly, a representative value of the parameter was selected within a range, such that the experimenter could easily adjust the difficulty according to the purpose of the experiment and achieve the purpose of the experimental situation.

## 5. NCAP test scenarios

### 5.1 Overview

In the section 4, the methodology of how to develop scenarios in this study was derived by benchmarking the PEGASUS project’s concept. This chapter contains examples of the process of constructing an actual test scenario according to the derived methodology. In this study, unlike the existing PEGASUS project, there is a difference in that a safety-in-use scenario based on traffic accident data was derived along with expert opinions.

### 5.2 Deriving dangerous situations using traffic accident data and expert opinions

The dangerous situations that AVs could face during lane keeping on a freeway were derived based on two types of data. The first used the descriptions from the KNPA freeway traffic accident data. The texts in the descriptions of the preprocessed 6,951 traffic accident data were classified and defined into eight categories, as shown in Table 3. The analysis revealed that the most common type was traffic accidents while driving at high speeds, accounting for 32.6% of the total. The "Not applicable traffic accidents" category shown in Table 3 denotes data corresponding to traffic accidents occurring during lane changing. The detailed accident situations were analyzed based on the individual categories to derive the possible dangerous situations that the AV could meet during lane keeping on freeways.

**Table 3:** Dangerous situations on freeways.

Dangerous Situations	No. of accidents	Rate (%)
Traffic accidents while driving at high speed	2,268	32.6
Traffic accidents while driving at low speed	246	3.5
Traffic accidents owing to stationary object	1,598	23.0
Traffic accidents owing to cut-in	829	11.9
Traffic accidents owing to cut-out	13	0.2
Traffic accidents caused by reverse vehicle	41	0.6
Other traffic accidents	716	10.3
Not applicable traffic accidents	1,240	17.8
Total	6,951	100

The second type of data reflected the opinions of freeway safety experts and AV experts. The freeway safety experts comprised the KEC employees who managed traffic at freeway sites, and researchers majoring in freeway safety management. The AV experts consisted of the Korea Automobile Testing & Research Institute (KATRI) researchers in charge of AV temporary driving permits and the NCAP, along with AV developers and AV testers. The expert opinions were used to derive dangerous situations, such as situations in which accident avoidance was expected to be difficult from an AV perspective, or threat situations in which it was difficult to avoid an accident even if a human driver was driving (as determined from an engineering perspective).

### 5.3 Functional scenarios

**Fig 2:**
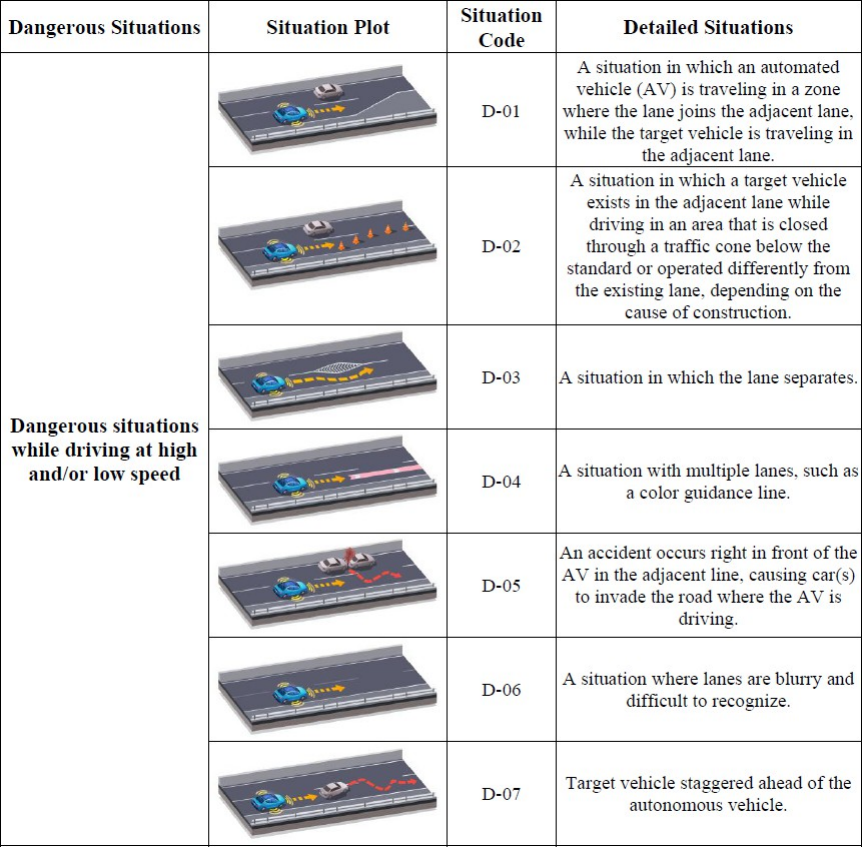
Functional scenarios for NCAP test(D-01 ~ D-07).

**Fig 3:**
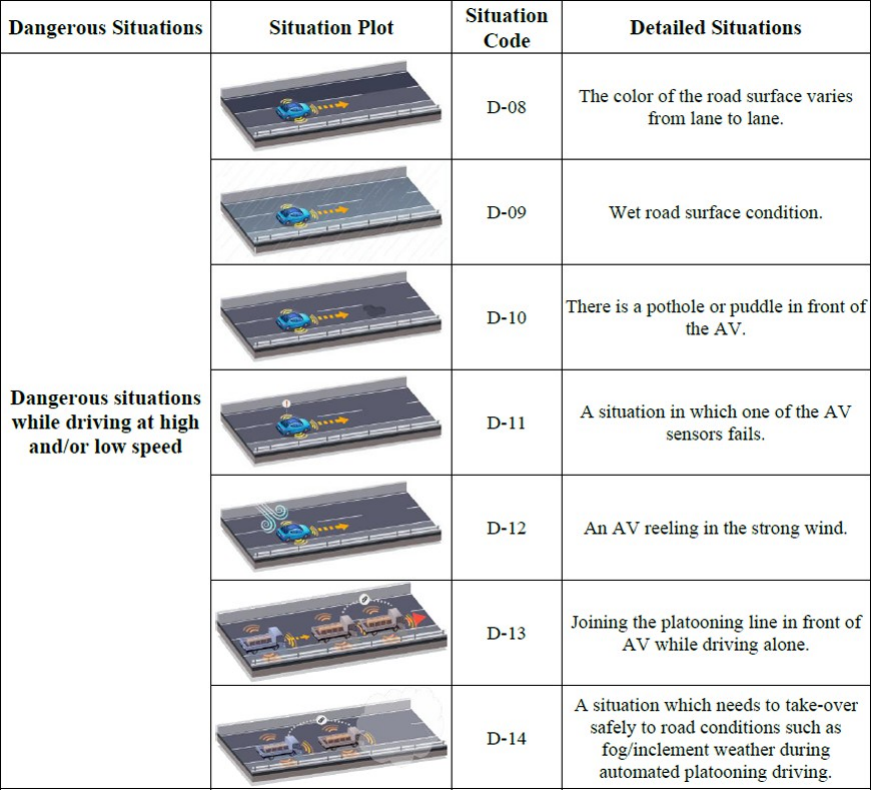
Functional scenarios for NCAP test(D-08 ~ D-14).

**Fig 4:**
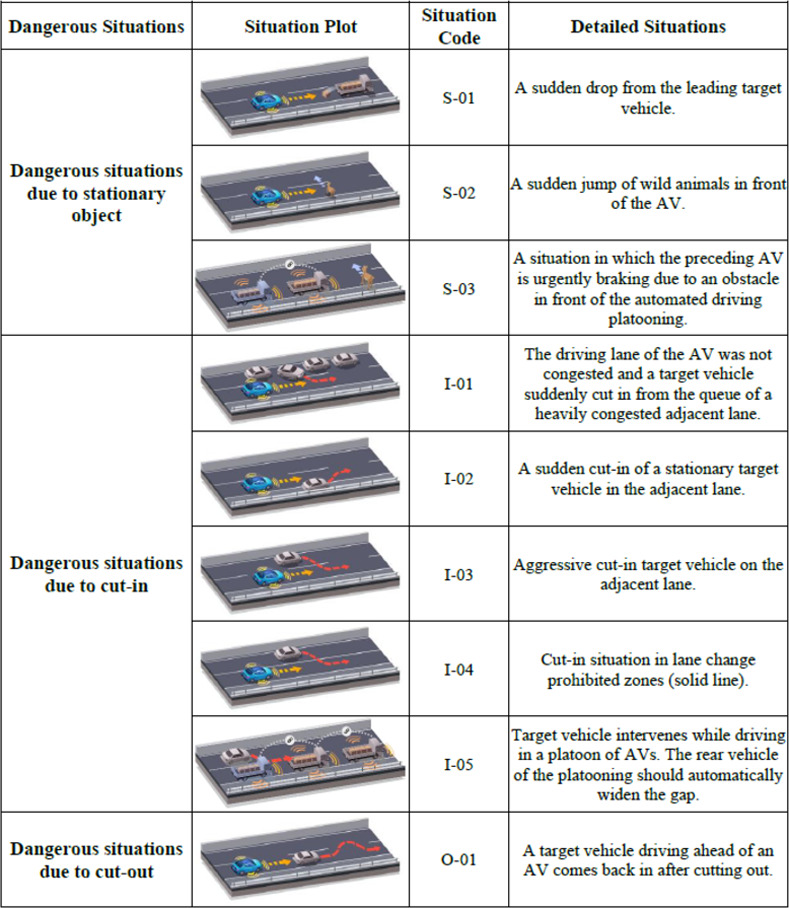
Functional scenarios for NCAP test(S-01 ~ O-01).

After a detailed analysis of specific situations during freeway driving, the functional scenarios for the NCAP test were finally developed by comparing them with the current enforcement regulations. The developed functional scenarios are presented from Figs 2–4. As can be seen from Figs 2–4., each scenario was differentiated from the enforcement regulations by targeting more dangerous situations, rather than general situations without any collisions. In the scenario codes, D represents a possible threat while driving, S represents a threat caused by a stationary object, and I and O are assigned to threats caused by a cut-in or cut-out, respectively; in addition, numbers are assigned sequentially for each situation.

### 5.4 Priority of test scenarios

In Table 4, The priorities of the previously developed functional scenarios were determined through the expert surveys and AHP. As a result of the expert surveys and AHP, the weights for each evaluation item derived from Table 2 are 0.12, 0.39, 0.19, 0.11, 0.19 respectively. The total score was calculated as shown in Table 5 by summing the derived weight multiplied by the respondent’s average score for each item. For example, the average score of respondents for each evaluation item in the ‘D-01’ situation is 4.25, 4.25, 2.50, 4.00, and 2.75, respectively. Here, the previously derived weights were multiplied. After multiplied, weighted score of evaluation items became 0.49, 1.66, 0.47, 0.45, and 0.54, respectively. Then, all the weighted values were added to obtain a total score of 3.60.

**Table 4:** Priorities of functional scenarios.

Priorities	Code	Detailed Situations	Total Score
1	I-01	The driving lane of the AV was not congested, and a target vehicle suddenly cut in from the queue of a heavily congested adjacent lane.	4.27
2	I-03	Aggressive cut-in target vehicle on the adjacent lane.	4.17
3	S-03	A situation in which the preceding AV is urgently braking due to an obstacle in front of the automated driving platooning.	4.16
4	D-09	Wet road surface condition.	4.15
5	D-05	An accident occurs right in front of the AV in the adjacent line, causing car(s) to invade the road where the AV is driving.	4.10
6	D-14	A situation which needs to take-over safely to road conditions such as fog/inclement weather during automated platooning driving.	4.06
7	D-11	A situation in which one of the AV sensors fails.	3.93
8	I-05	Target vehicle intervenes while driving in a platoon of AVs. The rear vehicle of the platooning should automatically widen gap.	3.90
9	D-02	A situation in which a target vehicle exists in the adjacent lane while driving in an area that is closed through a traffic cone below the standard or operated differently from the existing lane, depending on the cause of construction.	3.78
10	D-13	Joining the platooning line in front of AV while driving alone.	3.73
11	S-01	A sudden drop from the leading target vehicle.	3.71
12	D-06	A situation where lanes are blurry and difficult to recognize.	3.64
13	I-04	Cut-in situation in lane change prohibited zones (solid line).	3.63
14	S-02	A sudden jump of wild animals in front of the AV.	3.62
15	D-01	A situation in which an AV traveling in a zone where the lane joins the adjacent lane, while the target vehicle is traveling in the adjacent lane.	3.60
16	D-07	Target vehicle staggered ahead of the autonomous vehicle.	3.55
17	I-02	A sudden cut-in of a stationary target vehicle in the adjacent lane.	3.49
18	O-01	A target vehicle driving ahead of an AV comes back in after cutting out.	3.44
19	D-04	A situation with multiple lanes, such as a color guidance line.	3.44
20	D-10	There is a pothole or puddle in front of the AV.	3.38
21	D-12	An AV reeling in the strong wind.	3.29
22	D-08	The color of the road surface varies from lane to lane.	2.70
23	D-03	A situation in which the lane separates.	2.59

**Table 5:** Parameters and corresponding ranges.

Layers	Parameters		Ranges	Data sources
1 Road level	Design speed		100 km/h–120 km/h	Rules on the standards for structure and facilities of roads
	Lane width		More than 3.5 m	
	Minimum curve radius		Depends on design speed and superelevation (80 km/h: 280 m, 90 km/h: 380 m, 100 km/h: 480 m, 110 km: 600 m)	
	superelevation (cant)		Snow and cold areas in local areas: Max 6%,	
			The rest of local areas: Max 8%,	
			Urban areas: Max 6%,	
			Ramp: Max 8%	
	Cross slope		1.5%–2.0%	
	Number of lanes		1–5 lanes for one way	
	Maximum braking coefficient		0.3–1	Development of Autonomous vehicle Safety Evaluation Technology and Testbeds
2 Traffic infrastructure	Traffic facility		Bridge / Tunnel / Other / None	Freeway Public Data Portal (http://data.ex.co.kr/)
	Road marking type		White/Orange/Blue	Manual of traffic sign installation and management
			Solid/Dashed/doubled	
	Road marking standard		Dashed line painting 10 m	
			Lane spacing 10 m	
			Line width 0.10 m–0.15 m	
	Road marking reflection performance		White: Min 100 mcd/(m2 × Lux)	
			Yellow: Min 70 mcd/(m2 × Lux)	
			Blue: Min 40 mcd/(m2 × Lux)	
	Color lane		Presence / Absence	
3 Temporal modifications	bus lane		Presence / Absence	Road traffic law
	Dynamic hard shoulder		Presence / Absence	Freeway Public Data Portal (http://data.ex.co.kr
	Traffic cone		Presence / Absence	-
4 Objects	Number of vehicles required		Target: 1–n	-
			Ego: 1	
			Neighboring vehicle: 0–n	
	Initial headway distance		On demand	-
	Target vehicle	Vehicle speed	Stopped vehicle: 0 km/h,	Domestic insurance company
			Rapid braking vehicle: 80–110 km/h,	
			Low-speed vehicle: 40–55 km/h,	
			Stopped vehicle cut-in: 10–30 km/h	
		Acceleration	In case of a rapid braking vehicle: 5 m/s^2^	
		Lateral departure speed	Cut-in: 1.75 m/s–2.3 m/s	
		Distance between Ego	On demand	-
		Time-to-collision (TTC) with Ego	More than 0.72 s	Domestic insurance company
	Target object	Property	Metal / Other	Emergency automatic steering function evaluation criteria
		Size	Height × Width × Length = (Less than 3 mm) × 0.8 m × 2 m	
		Color	Degree of contrast to road surface	
	Ego (AV)	Vehicle speed	At high speed: 80–110 km/h,	Domestic insurance company
			At low speed: 30–50 km/h	
		Distance between Target vehicle	On demand	-
		TTC with Target	More than 0.72 s	Domestic insurance company
	Neighboring vehicle	Initial speed	Stopped vehicle: 0 km/h,	
			Rapid braking vehicle: 80–110 km/h,	
			Low-speed vehicle: 40–55 km/h	
		Distance from Ego	On demand	-
5 Environment	Lighting	Type	Day / Night	-
		Minimum ambient lighting	Day: 2,000–5,000	Development of Autonomous vehicle Safety Evaluation Technology and Testbeds
			Night: 500–2,000	
		Night road lighting rating	M1–M3	Road lighting criteria (Korean Standards Association)
		Road lighting brightness	M1: 2.00 (dry surface)	
			M2: 1.50 (dry surface)	
			M3: 1.00 (dry surface)	
			(In case of wet surface 0.15)	
		Road surface brightness in tunnel	60 km/h: 3–6	
			80 km/h: 5–8	
			100 km/h: 7–11	
			120 km: 7–11	
	Weather	Type	Sunny / Rain / Snow / Foggy	-
		Precipitation	0 mm–80 mm	Korea Meteorological Administration
		Maximum ambient temperature	Sunny: 5°C–40°C	Development of Autonomous vehicle Safety Evaluation Technology and Testbeds
			Rain: 5°C–40°C	
			Snow: -10°C–5°C	
		Minimum ambient temperature	Sunny: 5°C–40°C	
			Rain: 5°C–40°C	
			Snow: -10°C–5°C	
		Maximum wind speed	0 m/s–5 m/s	
6 Digital information	Sensor performance		Communication delay / Communication error / Position error / None	-

Finally, the situation with the highest priority was determined; it was a situation in which the driving lane of the AV was not congested and a target vehicle suddenly cut in from the queue of a heavily congested adjacent lane, with a total score of 4.27.

### 5.5 Selection of parameters and ranges for scenario

In this stage, the parameters and range of parameter values requiring consideration for all functional scenarios were selected. The corresponding parameters and values were necessary for the development of the logical and concrete scenarios. As for the range of parameters and values, the parameters and related data sources required for each layer defined in the PEGASUS project were investigated and analyzed, as shown in Table 5. In this study, digital information corresponding to Layer 6 was excluded from data collection. This was because specific cooperative intelligent transport systems services have not yet been defined in Korea.

### 5.6 Logical and concrete scenarios

According to the analysis above, the representative scenario with the highest priority was a situation in which the driving lane of the AV was not congested, and a target vehicle suddenly cut in from the queue of a heavily congested adjacent lane. It is difficult to derive various concrete scenarios for all of the functional scenarios. In this study, logical and concrete scenarios were constructed for the previously selected functional scenario. For the development of the logical scenario, the ranges were only set for the parameters related to the scenario among the previously selected parameters. In addition, the value of a parameter for creating a concrete scenario was derived based on the value most frequently used on average. Table 6 shows examples of logical and concrete scenario construction for the representative scenarios.

**Table 6:** Examples of logical and concrete scenarios.

Situations	The driving lane of the AV was not congested, and a target vehicle suddenly cut in from the queue of a heavily congested adjacent lane			
Layers	Logical Scenario			Concrete Scenario (Values)
	Parameters		Range	
1 Road level	Design speed		100 km/h–120 km/h	100 km/h
	Lane width		More than 3.5 m	3.5 m
	Cross slope		1.5%–2.0%	1.5%
	Number of lanes		1–5 lanes for one way	Two lanes for one way
	Maximum braking coefficient		0.3–1	0.9
2 Traffic infrastructure	Traffic facility		Bridge / Tunnel / Other / None	None
	Lane type		White/Orange/Blue	White solid
			Solid/dashed/doubled	
	Road marking standard		Dashed line painting 10 m	Dashed line painting 10 m
			lane spacing 10 m	lane spacing 10 m
			Line width 0.10 m–0.15 m	Line width 0.15 m
	Road marking reflection performance		White: Min 100 mcd/(m2 × Lux)	White: 150 mcd/(m2 × Lux)
			Yellow: Min 70 mcd/(m2 × Lux)	
			Blue: Min 40 mcd/(m2 × Lux)	
	Color lane		Presence / Absence	Absence
3 Temporal modifications	Bus lane		Presence / Absence	Absence
	Dynamic hard shoulder		Presence / Absence	Absence
	Traffic cone		Presence / Absence	Absence
4 Objects	Number of vehicles required		Target: 1	Depends on experimenters
			Ego: 1	
			Neighboring vehicle: 0–n	
	Initial headway distance		On demand	-
	Target vehicle	Vehicle speed	10–30 km/h	10 km/h
		Acceleration	Definition by experiment	-
		Lateral departure speed	Cut-in: 1.75 m/s –2.3 m/s	1.75 m/s
		Distance between Ego	On demand	-
		TTC with Ego	More than 0.72 s	0.72 s
	Ego (AV)	Vehicle speed	80–110 km/h	100 km/h
		Distance between Target vehicle	On demand	-
		TTC with Target	More than 0.72 s	0.72 s
	Neighboring vehicle	Initial speed	0–30 km/h	0 km/h
		Distance from Ego	On demand	-
5 Environment	Lighting	Type	Day / Night	Night
		Minimum ambient lighting	Day: 2,000–5,000	1,000 Lux
			Night: 500–2,000	
		Night road lighting rating	M1–M3	M3
		Road lighting brightness	M1: 2.00 (dry surface)	1.00 cd/ ㎡
			M2: 1.50 (dry surface)	
			M3: 1.00 (dry surface)	
			(In case of wet surface 0.15)	
		Road surface brightness in tunnel	60 km/h: 3–6	9 cd/㎡
			80 km/h: 5–8	
			100 km/h: 7–11	
			120 km: 7–11	
	Weather	Type	Sunny / Rain / Snow / Foggy	Sunny
		Maximum ambient temperature	Sunny: 5°C–40°C	40°C
			Rain: 5°C–40°C	
			Snow: -10°C––5°C	
		Minimum ambient temperature	Sunny: 5°C–40°C	5°C
			Rain: 5°C–40°C	
			Snow: -10°C––5°C	
		Maximum wind speed	0 m/s–5 m/s	5 m/s
6 Digital information	Sensor performance		Communication delay / Communication error /Position error / None	None

An example of a concrete scenario was presented earlier. In fact, for individual functional scenarios, many concrete scenarios can be created to describe various situations by changing the parameter values in the logical scenario within the corresponding range, or by changing the locations of the target vehicles or neighboring vehicles. The work of generating such a concrete scenario can be conducted according to a systematic procedure; alternatively, such a scenario can be created by setting values at random within the parameter ranges of the logical scenario. The latter case is thought to be frequently used in simulation-based tests using vehicle-in-the-loop or hardware-in-the-loop simulations. In this case, it is necessary to select valid values by considering the relationships between parameters to perform a meaningful experiment. A scenario in which the range of effective values ​​is reduced after considering the relationships with other parameters is called a test case. The process of creating a concrete scenario in this way may vary depending on the experimental method, such as whether it is based on a proving ground, real road, or simulation.

## 6. Conclusions

With the introduction of AV approaches, there is a need to provide information on the safety of AVs to people who want to purchase AVs. Vehicle safety information is currently provided through NCAPs. An NCAP encourages car manufacturers to develop safety technologies and, through evaluation results, contributes to helping consumers choose a safer car when purchasing a car. However, as the KNCAP currently only provides safety information for general vehicles equipped with ADASs, it will be necessary to provide safety information for AVs in the near future.

In this study, a methodology was established for deriving NCAP test scenarios for providing AV safety information. Prior to establishing the methodology, the necessity and direction of the AV NCAP test were established. In Korea, safety standards and enforcement regulations related to maintaining lanes on freeways have been developed for Level 3 AVs. Accordingly, it was determined that a more challenging assessment should be conducted to differentiate from the NCAP test. Therefore, the AV test scenario of this study was derived based on an assessment of situations in which the AV responds when another vehicle is driving. The NCAP evaluation scenario referred to the format of the PEGASUS project to establish an internationally compatible and sustainable assessment methodology. A sustainable assessment methodology was constructed by gradually specifying the scenario, and the variables necessary for the experiment were divided into six layers to secure systematization. In this study, the detailed situations of general automobile accident data were classified into eight categories. By reflecting the detailed classifications of accident data and opinions of experts, it was possible to derive the dangerous situations for AVs, and to build a functional scenario. After deriving a functional scenario, it was considered that the evaluation scenario construction is sustainable, based on verification using the AHP and the derived priorities. In addition, it was determined that the variables of the logical and concrete scenarios could be used continuously, e.g., through continuous updates.

## Supporting information

S1 File (ZIP)
